# Genomic surveillance of SARS-CoV-2 in patients presenting neurological manifestations

**DOI:** 10.1371/journal.pone.0270024

**Published:** 2022-06-30

**Authors:** Anna Vicco, Francesca Caccuri, Serena Messali, Adriana Vitiello, Aron Emmi, Claudia Del Vecchio, Alberto Reale, Arnaldo Caruso, Giancarlo Ottaviano, Carla Mucignat, Cristina Parolin, Angelo Antonini, Arianna Calistri

**Affiliations:** 1 Department of Molecular Medicine, University of Padua, Padua, Italy; 2 Department of Molecular and Translational Medicine, Section of Microbiology, University of Brescia, Brescia, Italy; 3 Department of Neuroscience, University of Padua, Padua, Italy; 4 Microbiology and Virology Unit, Padua University Hospital, Padua, Italy; 5 Department of Neuroscience, Otolaryngology Section, University of Padua, Padua, Italy; 6 Parkinson and Movement Disorders Unit, Study Center for Neurodegeneration (CESNE), Department of Neurosciences, University of Padua, Padua, Italy; Cairo University, EGYPT

## Abstract

During the first wave of infections, neurological symptoms in Coronavirus Disease 2019 (COVID-19) patients raised particular concern, suggesting that, in a subset of patients, the severe acute respiratory syndrome coronavirus 2 (SARS-CoV-2) could invade and damage cells of the central nervous system (CNS). Indeed, up to date several *in vitro* and *in vivo* studies have shown the ability of SARS-CoV-2 to reach the CNS. Both viral and/or host related features could explain why this occurs only in certain individuals and not in all the infected population. The aim of the present study was to evaluate if onset of neurological manifestations in COVID-19 patients was related to specific viral genomic signatures. To this end, viral genome was extracted directly from nasopharyngeal swabs of selected SARS-CoV-2 positive patients presenting a spectrum of neurological symptoms related to COVID-19, ranging from anosmia/ageusia to more severe symptoms. By adopting a whole genome sequences approach, here we describe a panel of known as well as unknown mutations detected in the analyzed SARS-CoV-2 genomes. While some of the found mutations were already associated with an improved viral fitness, no common signatures were detected when comparing viral sequences belonging to specific groups of patients. In conclusion, our data support the notion that COVID-19 neurological manifestations are mainly linked to patient-specific features more than to virus genomic peculiarities.

## Introduction

The severe acute respiratory syndrome coronavirus 2 (SARS-CoV-2) is a member of the Coronaviridae family, a group of viruses that can infect both mammals and birds. SARS-CoV-2 has a probable zoonotic origin with bat as primary host [1, 2]. Differently from endemic human Coronaviruses, e.g. OC43 and 229E viruses, SARS-CoV-2 not only infects the upper respiratory tract, but it can also spread to the lower tract, leading to a more severe respiratory disease [3].

SARS-CoV-2 genome is a positive-sense single-stranded RNA that contains 14 open reading frames (ORFs). At the 5’ end of the genome, there are the leader sequence and an untranslated region (UTR). Then, the gene encoding for the viral Replicase-Transcriptase complex, which is an RNA-dependent RNA polymerase, occupies two-thirds of the genome. This gene is made of two ORFs, ORF1a and ORF1b, that are translated together as two co-terminal polyproteins [4]. These polyproteins then auto-proteolytically cleave themselves into several products called non- structural proteins (nsp from 1 to 16) with several functions. One of the most important nsp is the nsp12 that represents the RNA-dependent RNA polymerase (RdRp). At the 3’ end of the genome instead are located the genes encoding for the following structural proteins: i) the spike S glycoprotein, ii) the envelope E protein, iii) the membrane M protein, iv) the nucleocapsid N protein. Genes encoding for accessory proteins are also located at the 3’ terminal. In the viral particle, the RNA genome is tightly bound to N in a helical symmetric nucleocapsid. The viral particle is surrounded by an envelope made of lipid layers acquired from the host Endoplasmic Reticulum-Golgi intermediate compartment (ERGIC) and containing the viral spike (S), envelope (E) and membrane (M) proteins [5]. Viral RNA genome is known to rapidly acquire mutations while replicating, as RNA-dependent RNA polymerases are more prone to introduce errors than DNA-dependent DNA polymerases. Interestingly, the replicase of *Coronaviridae* is characterized by a proofreading activity linked to the amino-terminal exoribonuclease (ExoN) domain of the Nsp14 enzyme that can correct errors inserted in the viral genome [6]. Although this feature reduces the rate of sequence variability, SARS-CoV-2 can still acquire relevant mutations as it spreads worldwide. Indeed, the constant and relevant human-to-human transmission, the natural adaptation of the virus to the new host as well as the more recent pressure of vaccination, facilitate occurrence of mutations that can be fixed in the viral population, as shown by the emergence of different variants since the beginning of the pandemic (https://www.cdc.gov/coronavirus/2019-ncov/variants/variant-classifications.html, accessed on 17^th^ of May 2022). Currently, viral variants are classified into larger groups named lineages or clades. Different clade/lineage nomenclatures have been proposed. Among these, the Phylogenetic Assignment of Named Global Outbreak Lineages (PANGOLIN) is a software that allows a dynamic nomenclature (known as the PANGO nomenclature) of SARS-CoV-2 lineages [7]. SARS-CoV- 2 variants are grouped according to their lineage and component mutations [8]. The World Health Organization (WHO) classifies some of the emerging variants as variants of concern (VOC), variants of interest (VOI) or variants under monitoring (VUM), based on their transmissibility and/or infection severity. All these variants are named with letters of the Greek alphabet [9]. Delta (B.1.617.2) and Omicron (B.A.1 and B.A.2) are the VOC currently circulating (https://www.ecdc.europa.eu/en/covid-19/variants-concern, accessed on 12^th^ of May 2022).

Viral entry into host cells is mainly mediated by the S envelope protein, which is composed of two subunits named S1 and S2 [10]. Virus attachment to the target cells involves S1 and the host angiotensin-converting enzyme 2 (ACE-2) receptor. Following steps are allowed by the cellular proteins cathepsin L and transmembrane protease serine 2 (TMPRSS2) that, acting on the S1/S2 complex, lead to the exposure of a fusion peptide belonging to the S2 subunit [10].

Coronavirus Disease 2019 (COVID-19) is mainly a respiratory disease, but it can also involve additional organs, causing hepatic, enteric, neurological as well as psychiatric symptoms [11]. Neurological manifestations associated to COVID-19 have attracted particular attention. Among the most frequent neurological signs detected in COVID-19 patients there are headache, dizziness, nausea, confusion [12, 13], smell and taste disorders even at early stages of the disease [14, 15], while the most common complications are stroke, neurological damage, ataxia, delirium, brain and spinal cord inflammation [13], encephalopathies and encephalitis [16, 17]. Moreover, an increased incidence of Acute Disseminated EncephaloMyelitis (ADEM) has been reported in COVID-19 patients [18]. Different respiratory viruses, including Coronaviruses, are known to infect the brain tissue [19, 20]. For example, neuroinvasion and neurovirulence of the human coronavirus OC43 (HCoV-OC43), SARS-CoV-1 and Middle East Respiratory Syndrome (MERS)-CoV were reported in transgenic mice [20, 21]. Importantly, ACE2 was shown to be highly expressed in neurons, astrocytes, and oligodendrocytes [22, 23]. The SARS-CoV-2 ability to infect the human brain has been further analyzed in human neural progenitor cells and brain organoids [24, 25]. In addition, SARS-CoV-2 RNA was detected in brains [26] and in the cerebrospinal fluid (CSF) of some COVID-19 patients, indicating a possible pathophysiological involvement of the virus in the neurological symptoms [27]. The mechanisms accounting for SARS-CoV-2 related neurological manifestations have been deeply analyzed in many recent studies (reviewed in [11]). Up to now, two hypotheses have been proposed to explain the COVID-19 neuropathogenesis. On the one hand, SARS-CoV-2 may enter the CNS through the olfactory bulb by binding to the ACE-2 receptor, damaging the blood brain barrier (BBB) or even exploiting leukocytes as a Trojan horse mechanism [28]. The neurological symptoms would be, then, a consequence of the direct viral damage to brain tissues. In this context, specific regions of the viral genome could play a role in neurotropism and/or neurovirulence. On the other hand, SARS-CoV-2 replication in the lungs, in addition of causing pulmonary dysfunctions, is known to trigger an intense and dysregulated systemic inflammatory process called cytokine storm, which is a common feature of severe COVID-19 cases. The cytokine storm has consequences for the entire organism and might affect the CNS, even without a direct invasion of the brain by the virus [28, 29]. Finally, viral entry into the CNS may trigger a localized inflammatory response that could have an impact on the CNS by itself, or in combination with the cytokine storm [11, 29].

Here we investigated whether viral genomes obtained from selected patients displayed peculiar mutations associated to the degree of severity of the neurological symptoms. To avoid artifacts due to the high viral mutation rates *in vitro*, we avoided virus amplification in cell culture and directly sequenced the viral genomic RNA obtained from the nasopharyngeal swabs of the enrolled individuals. First, we focused our attention on two SARS-CoV-2 envelope proteins: i) the spike glycoprotein, which is responsible for the viral tropism and the viral entry into target cells, ii) the E protein, that is linked to the modulation of the inflammatory responses in several Coronaviruses. Second, a whole genome analysis was performed to detect mutations that might be related to neurovirulence. Overall our data indicate that no mutations can be found associated to specific neurological symptoms or to their severity, thus supporting the notion that CNS manifestations in COVID-19 patients are mainly linked to the individual inflammatory response, more than to peculiar viral features.

## Materials and methods

### Study design

The study was approved by the Padua Hospital Research Ethics Committee (Protocol 056881). A group of patients displaying SARS-CoV-2 related neurological symptoms with different degrees of severity was selected, as shown in Table 2 (Results section). All enrolled subjects underwent Magnetic Resonance Imaging (MRI). Informed consent was obtained from all participants.

Nasopharyngeal swabs were handled, using standard procedure to inactivate the virus. Viral RNA was directly extracted by an automatic nucleic acids extractor (MagNA Pure by Roche), following the manufacturer’s instructions. Obtained samples were coded with letters from A to G, according to chronological order of the extraction date. In particular, viral extracts from patients A, B, C, D, E and F were collected between March 4 and 27, while the viral RNA from patient G was extracted on November 23, 2020. Following analyses were conducted in blind, meaning that no information on symptoms were known until the overall sequencing results were analyzed.

### Sanger sequencing

**Fig 1:**
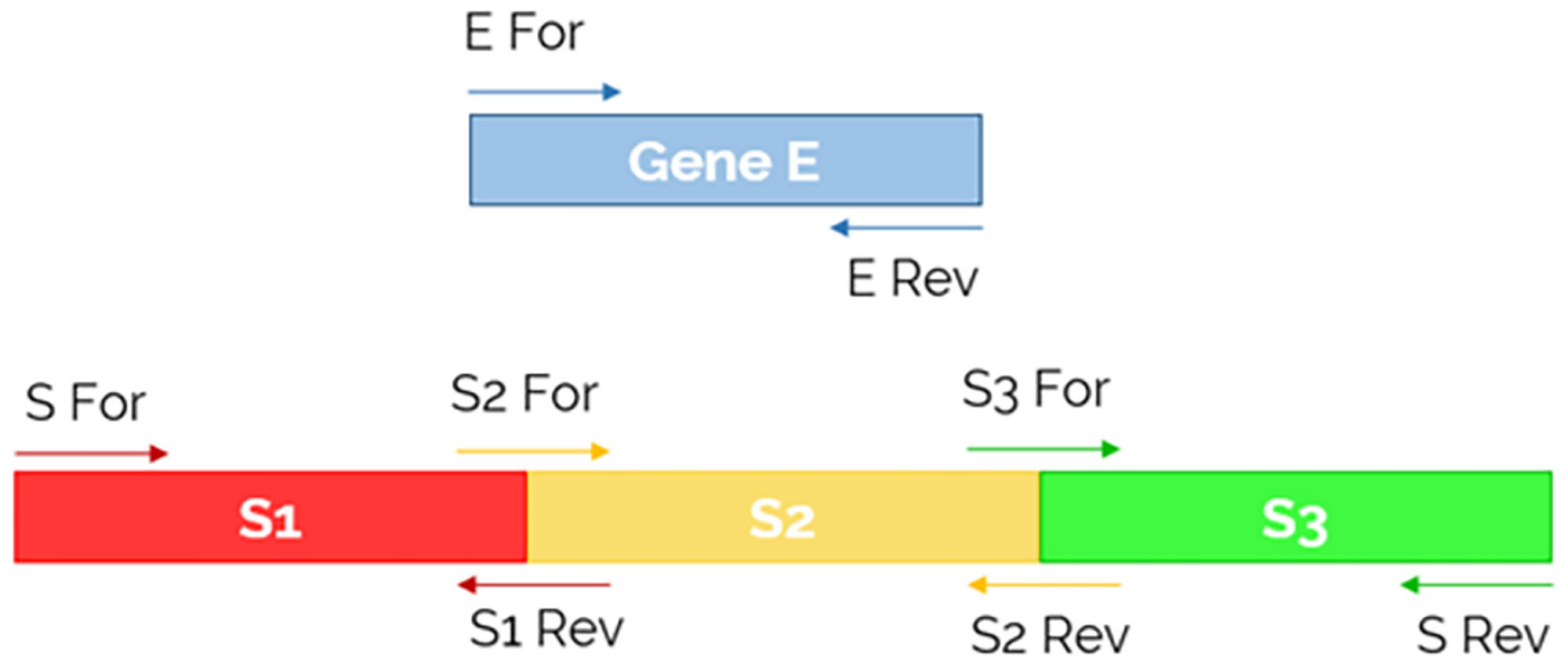
Primer position within the gene of interest. The Fig schematically display where primers adopted for reverse-transcription, amplification and sequencing of the E (light blue) and S (red, yellow and green) genes map within the viral regions of interest. While a single pair of oligonucleotides were designed to amplify and sequence the entire E gene, one couple of external primers along with two additional pairs of internal primers were used in the case of the S gene. “E For” stands for primer E forward; “E Rev” for primer E reverse, “S For” for primer S forward; “S1 Rev” for primer S1 reverse; “S2 For” for primer S2 forward; “S2 Rev” for primer S2 reverse, “S3 For” for primer S3 forward; “S Rev” for primer S reverse.

First, genes of interest (E and S) were retro-transcribed from total RNA viral extracts by the AgPath-IDT One-Step RT-PCR kit (Thermofisher), following the manufacturer’s instructions. The adopted oligonucleotides sequences and position within the target are reported in Table 1 and in Fig 1, respectively. Briefly, for the E sequence adopted oligonucleotides mapped at the 3’ and 5’ ends of the gene; while three pairs of primers were designed to reverse-transcribe the large S gene into three fragments of similar length (Fig 1).

**Table 1:** Sequences of primers adopted for Sanger sequencing of E and S genes.

Primer name	Primer sequence 5’-3’
Primer E forward	ATGTACTCATTCGTTTCGGA
Primer E reverse	TTAGACCAGAAGATCAGGA
Primer S forward	ATGTTTGTTTTTCTTGTTTTATTGCCACTA
Primer S reverse	GCTCAAAGGAGTCAAATTACATTACACATA
Primer S1 reverse	GGGCAAACTGGAAAGATTGCTG
Primer S2 forward	GCAAACTGGAAAGATTGCTGATTATA
Primer S2 reverse	TCTACTTTTCAACAAAGTGACACTTG
Primer S3 forward	TTTCAACAAAGTGACACTTGCAGAT

The table displays the sequences of the oligonucleotides adopted for reverse-transcription and amplification of the S and E genes performed in order to Sanger sequence these viral genomic regions.

Next, to obtain a sufficient amount of cDNA, an additional PCR amplification step was carried on by using the same primers described above along with the high-fidelity DNA-dependent polymerase Phusion Hot Start II High-Fidelity DNA Polymerase (Invitrogen). The PCR products were purified by NucleoSpin Gel and PCR Clean-up kit (Macherey-Nagel), and 6.4 pmoles were dried at 65°C for a few minutes. Sanger sequencing of the PCR products was performed by BMR Genomics (Padua, Italy), by adopting the same pairs of oligonucleotides reported in Table 1 and Fig 1.

### Whole genome sequencing

For the whole genome sequencing, an amplicon-based approach, targeting 343 partially overlapping subgenomic regions, that cover the entire SARS-CoV-2 genome, was used. Virus genomes were generated using Paragon Genomics’ CleanPlex multiplex PCR Research and Surveillance Panel, according to the manufacturer’s protocol [30, 31]. Briefly, similar amounts of RNA were reverse transcribed with random primers and the resulting cDNA, magnetically purified, was used as template in a 10 μl-multiplex PCR performed with two pooled-primer mixtures. Samples were treated with 2 μl of CleanPlex digestion reagent at 37°C for 10 min to remove non-specific PCR products. After magnetic bead purification, PCR products were subjected to further 25 rounds of amplification in a secondary PCR where indexed primers allow to generate the amplicons library. Subsequently, purified libraries were quantified with the Qubit DNA HS Assay Kit (Thermo Fisher Scientific). Amplicon libraries were loaded in a 300-cycle sequencing cartridge and deep sequencing was performed on MiSeq platform (Illumina, San Diego, CA, USA). Sequencing raw data were checked for quality using FastQC (https://www.bioinformatics.babraham.ac.uk/projects/fastqc) and then analyzed with software SOPHiA GENETICS’ SARS-CoV-2 Panel (SOPHiA GENETICS, Lausanne, Switzerland). To confirm data analyses, the paired-end reads were trimmed with Trimmomatic ver. 0.38 for quality (Q score > 25) and length (> 36 bp) and assayed by the Geneious^®^ software (version 11.1.5) (Biomatters Ltd, New Zealand). The consensus sequence was reconstructed by mapping the reads to the SARS-CoV-2 reference sequence NC_045512 using Bowtie2 in sensitive-local mode with consensus threshold at 65%. The variant calling was carried out by the Variant Finder Tool (Geneious) filtering out variants with a p-value greater than 0, using a minimum variant frequency of 0 and default parameters for Maximum Variant p-value (10^−6^).

### Sequence alignment and classification of viral sequences into SARS-CoV-2 lineages and clades

The pool of sequences obtained by NGS was aligned with those reported in the GISAID online database (https://www.gisaid.org/ first access on 15^th^ of March 2021). Specifically, mutation analysis was performed exploiting the CoVsurver application of GISAID (https://www.gisaid.org/epiflu-applications/covsurver-mutations-app/ last accessed on the 17^th^ of May 2022). Classification into clades was performed through Nextstrain (https://nextstrain.org/SARS-CoV-2/ accessed on the 15th of March 2021), while lineage assessment was conducted using the Phylogenetic Assignment of Named Global Outbreak LINeages (PANGOLIN) tool available at https://github.com/hCoV-2019/pangolin [32] (accessed on the 15^th^ of March 2021).

### Phylogenetic analysis

Public SARS-CoV-2 complete genome sequences (>29 Kb), available in the GISAID repository until December 2021 were retrieved. Low-quality genomes and nearly identical sequences (genetic similarity > 99.99%) were excluded, obtaining a global dataset of 2463 public genomes plus 7 genomes reported in this study. The global datasets of 2470 whole genome sequences were aligned by MAFFT (FF-NS-2 algorithm) using default parameters [33]. The alignment was manually curated using Aliview [34] to remove artifacts at the ends. Phylogenetic analysis was performed using IQ-TREE (version 1.6.10) under the best fit model according to Bayesian Information Criterion (BIC) indicated by the Model Finder application implemented in IQ-TREE [35]. The statistical robustness of individual nodes was determined using 1000 bootstrap replicates.

## Results

### The S and E proteins shared a conserved sequence among the SARS-CoV-2 genomes under study

Patients enrolled in this study were all SARS-CoV-2 positive subjects belonging to one of the following three groups based on their neurological symptoms: symptomatic, mild-symptomatic, asymptomatic. Patients were coded with letters from A to G, according to chronological order of viral RNA extraction date. In this manuscript, for clarity reasons, these capital letters are followed by the lower case letters “s”, “m” or “a”, representing the relative neurological symptomatology (symptomatic, mild-symptomatic, asymptomatic, respectively). In more details:

- Patients As, Fs and Gs were severely symptomatic (s), displaying neurological signs already reported as associated to COVID-19, i.e. generalized seizures and encephalopathy. In addition to these common manifestations, patient Fs had also a stroke. As, Fs, Gs were hospitalized and displayed severe respiratory symptoms, requiring intubation.
- Patients Cm and Em were mildly symptomatic (m), showing smell deficit, that was full recovered only by patient Em.
- Patients Ba and Da were the negative controls, as they were SARS-CoV-2 positive but display neither neurological nor non-neurological symptoms [asymptomatic (a)].

None of the patients reported relevant comorbidities. Main information on the enrolled individuals is summarized in Table 2.

**Table 2:** Characteristics of the enrolled COVID-19 patients relevant for the study.

Code	Age	Sex	Clinical symptoms	Imaging	Outcome
As	55	M	Encephalopathy, generalized seizures and interstitial pneumonia	MRI negative	Full recovery
Ba	29	M	Asymptomatic	MRI negative	Full recovery
Cm	26	F	Smell deficit	MRI negative	Smell dysfunction after 6 months
Da	30	F	Asymptomatic	MRI negative	Full recovery
Em	31	F	Smell deficit	MRI negative	Full recovery
Fs	74	M	Encephalopathy, seizures, stroke and interstitial pneumonia	Lesions related to stroke	Hemiparesis, cognitive disturbances
Gs	77	F	Encephalopathy, generalized seizures and interstitial pneumonia	MRI negative	Full recovery

The Table reports main information of COVID-19 patients enrolled in this study. Patients were coded with capital letter from A to G. In this manuscript, additional lower case letters were associated to each patient in relation to her/his neurological symptoms as follows: s = symptomatic, m = mild-symptomatic, a = asymptomatic. Each patient underwent magnetic resonance imaging (MRI).

Total viral RNA was recovered directly from nasopharyngeal swabs of the selected patients, to avoid possible accumulation of mutations during SARS-CoV-2 amplification in cell culture. Next, the S and E envelope genes were Sanger sequenced. In the case of the S gene, all sequences were sharing the same A to G substitution with respect to the Wuhan-Hu-1 reference sequence (NCBI Reference Sequence: NC_045512.2), at position 23403 of genomic RNA. This nucleotide variation corresponds to a predicted amino acidic substitution of an aspartic acid (D) to a glycine (G) in the amino acidic position 614. The D614G is well known as it represents the most significant characteristic of an early SARS-CoV-2 variant [36] that rapidly spread in Europe and USA, becoming responsible for the greatest number of infections, by May 2020. Our data show that this variant was circulating in Italy (Veneto Region), since early March 2020. In the case of the E gene, all sequences were identical to the one of the Wuhan reference strain.

In conclusion, from viral genomes deriving from a specific group of patients no peculiar and common sequence signature were detected in the S or E genes, at least with the Sanger sequencing.

### Whole genome sequencing highlighted several known mutations present in the viral extracts but none common and unique of SARS-CoV-2 genomes retrieved from neurological symptomatic patients

Despite the finding that all viral genomes under evaluation shared identical E and S genes, peculiar features associated with neurological symptoms could not be ruled out in other SARS-CoV-2 genomic regions. Thus, a whole-genome analysis by next generation sequencing was carried out starting from the same viral RNA extracts adopted for the E and S Sanger sequencing. Specifically, the Illumina MiSeq platform was applied on 343 partially overlapping subgenomic regions, obtained by an amplicon-based approach to cover the entire SARS-CoV-2 genome. Obtained sequences were aligned with the Wuhan-Hu-1 reference sequence (NCBI Reference Sequence: NC_045512.2). The Geneious Find Variations/SNPs program, used to evaluate the presence of mutations in our samples, finds variants above a minimum threshold to screen out disagreements due to read errors. The tool calculates p-values for variations and filters only the single-nucleotide variations (SNVs) with a specified maximum P-Value. The lower the p-value, the more likely the variation at the given position represents a real variant. For this reason, we decided to filter out variants with a maximum p value of 10^−6^, with 0.001% probability to see variant by chance. We selected stringent parameters, together with a minimum coverage of 10 reads, to have the possibility to find all the SNVs present in patient’s samples. Nevertheless, almost all of the identified mutations have a depth of more than 100 reads with a variant frequency >70%.

Found mutations are reported in Table 3. Among the most significant ones, 4 mutations (genomic positions 241, 3037, 14408 and 23403 of the RNA letter code), characteristic of lineage B.1, were found in all the analyzed viral genomes. Another set was shared between the viral extract obtained from 1 mild symptomatic patient (Cm) and 1 asymptomatic subject (Ba). This was a variation of 3 nucleotides at position 28881-2-3, which is a main feature of most sequences belonging to Lineage B.1.1.

**Table 3:** Summary of the mutations retrieved in each extract by whole genome analysis.

Pos	Ref	Mut	Gene	Protein	Type	As	Ba	Cm	Da	Em	Fs	Gs
241	C	T	5’UTR		5’UTR	T	T	T	T	T	T	T
626	G	T	ORF1ab	leader	Non-syn	WT	V121F	WT	WT	WT	WT	WT
3037	C	T	ORF1ab	nsp3	Syn	F924	F924	F924	F924	F924	F924	F924
8334	C	T	ORF1ab	nsp3	Non-syn	A2690V	WT	WT	WT	WT	WT	WT
11652	T	C	ORF1ab	nsp6	Non-syn	L3796P	WT	WT	WT	WT	WT	WT
12153	C	T	ORF1ab	nsp8	Non-syn	WT	WT	A3963V	WT	WT	WT	WT
14408	C	T	ORF1ab	RdRp	non-syn	P4715L	P4715L	P4715L	WT	P4715L	P4715L	P4715L
18417	T	C	ORF1ab	nsp14	syn	D6051	WT	WT	WT	WT	WT	WT
23403	A	G	S	S	Non-syn	D614G	D614G	D614G	D614G	D614G	D614G	D614G
25433	C	T	ORF3a		Non-syn	T14I	WT	WT	WT	WT	WT	WT
25459	G	T	ORF3a		Non-syn	WT	A23S	WT	WT	WT	WT	WT
28881	G	A	N	N	Non-syn	WT	R203K	R203K	WT	WT	WT	WT
28882	G	A	N	N	Non-syn	WT	R203K	R203K	WT	WT	WT	WT
28883	G	C	N	N	Non-syn	WT	G204R	G204R	WT	WT	WT	WT
28932	C	T	N	N	Non-syn	WT	WT	WT	WT	WT	A220V	A220V
29568	T	C	ORF10		Non-syn	I4T	WT	WT	WT	WT	WT	WT

The Table reports in the first column the position (Pos) of the mutated nucleotide within the viral genome. Ref stands for the nucleotide located in Pos within the reference sequence, Mut for the mutated nucleotide, Gene and Protein for the gene and the protein where the mutation is located, respectively, Type for the type of mutation, Syn for synonymous mutation, Non-syn for non-synonymous mutation, WT for wild-type when identical to the reference sequence, mut for mutated when compared to the reference sequence, From column #7 to column #13 the amino acidic changes are reported when appropriate.

Among patients characterized by the most severe clinical situation, the viral RNA extracted from patients Fs and Gs shared C28932T substitution in the RNA sequence. This mutation causes the change of an alanine (A) to an amino acid of similar properties, valine (V), in position 220 of the amino acidic sequence (A220V). The A220V non-synonymous mutation is located within the viral nucleocapsid N protein. The alignment with sequences present in the Global Initiative on Sharing All Influenza Data (GISAID) repository until the 17^th^ of May 2022 showed that the A220V mutation has a frequency of roughly 1.6% (Table 4). A220V has been reported in viral genomes from 113 Countries, with the first one reported in February 2020 in Morocco, and predominant in Spain during summer 2020. Interestingly, A220V displays a distribution pattern similar to the one of the A222V of the S glycoprotein [37]. In fact, both mutations are generally associated in the 20A.EU1 clade. However, both Fs and Gs viral extracts lack the A222V change. Interestingly, while the Fs nasopharyngeal swab was performed in early March 2020, the Gs was obtained in November of the same year. Of note, the third severely symptomatic patient (As) lacks the A220V polymorphism.

**Table 4:** Highlights on the results of the sequence alignment with those retrieved in the GISAID repository.

Pt	Nt	Gene	Protein	Type	aa change	Freq	N_seq	N_Countries	First Country	Date (2020)
**As**	C8334T	ORF1ab	nsp3	Non-syn	A2690V	0.05%	5754	77	Italy	March
**As**	T11652C	ORF1ab	nsp6	Non-syn	L3796P	0.00%	52	15	Italy	April
**As**	T18417C	ORF1ab	nsp14	Syn	D6051	NC	/	/	/	/
**As**	C25433T	ORF3a	nsp3	Non-syn	T14I	0.08%	7961	90	France	March
**As**	T29568C	ORF10	/	Non-syn	I4T	NC	/	/	/	/
**Ba**	G626T	ORF1ab	leader	Non-syn	V121F	0.02%	2005	56	China	Feb
**Ba**	G25459T	ORF3a	nsp3	Non-syn	A23S	0.11%	11614	99	Netherlands	Feb
**Cm**	C12153T	ORF1ab	nsp8	Non-syn	A3963V	0.05%	4992	81	Italy	March
**Fs + Gs**	C28932T	N	N	Non-syn	A220V	1.63%	171532	113	Morocco	Feb

The Table reports some of the results obtained by the alignment of the sequences obtained in this study with those present in the GISAID repository until the 17^th^ of May 2022. In particular, the table focuses on those mutations highlighted by the sequencing alignment that were not previously described in literature. Pt stands for patient, nt for the mutated nucleotide, Gene and Protein for the gene and the protein where the mutation is located, respectively, Type for the type of mutation (i.e. Non-syn for non-synonymous, Syn for synonymous), aa change for the amino acidic mutation, N_seq for the number of sequences with the mutation, N_Countries corresponds to the number of Countries that had reported the mutation, and the first to report it (First Country), Date stands for date of database entry, NC (non-classified), "/" (not specified).

In addition to known mutations, viral genomes obtained from patients As (symptomatic), Ba (asymptomatic) and Cm (mild-symptomatic) displayed “extra” polymorphisms that are less studied. When sequences were aligned with those reported in the online GISAID database until the 17^th^ of May 2022 (Table 4) these extra polymorphisms were characterized, in general, by a frequency below 1%, with the nucleotide substitution G25459T (patient Ba), approaching 0.1%, and the T11652C (patient As), approaching 0%. Among the mutations reported in Table 4 the most interesting are the following:

- C25433T in ORF3a of viral extract As: in the amino acidic code this mutation is translated into T14I, with a reported frequency of around 0.08%. This mutation was shared by 7961 sequences present in the GISAID database (until May 2022), reported in 90 Countries with the first sequence dating back March 2020. This substitution changes a polar amino acid, threonine (T), to a non-polar one, isoleucine (I).
- T29568C in ORF10 of viral extract As: it leads to an amino acidic substitution from a non-polar amino acid, isoleucine (I), to a polar one, threonine (T), i.e. I4T. GISAID analysis (May 2022) did not find sequences with such a mutation reported in the database. However, it is likely that, in a genome 29.9 kb-long, a mutation located in position 29600 is in a non-coding region.
- G25459T in ORF3a of viral extract Ba: it is translated in an amino acid change from a non-polar amino acid, alanine (A) to a polar one, serine (S), and named A23S. It has a frequency of 0.11% and has been reported in 11614 sequences of 99 Countries (GISAID May 2022), with the first sequence deposited in February 2020 in Netherlands.
- T18417C in ORF1ab of viral extract As: it is the only synonymous mutation reported in patient A. This mutation is located in the nsp14, the protein responsible for the proof-reading activity of the polymerase.

It must be noted that there were no sequences reported in the online database with 100% similarity to the sequences obtained from patients As, Ba and Cm, at least up to May 2022.

### Classification of the identified SARS-CoV-2 genomes into clades and lineages and phylogenetic analysis

Based on the NGS analysis, viral sequences were classified in their corresponding lineages by adopting the Phylogenetic Assignment of Named Global Outbreak LINeages (PANGOLIN) tool. In more details, the results showed that viral sequences from patients As, Da and Em belonged to B.1 lineage, while the ones from patients Fs and Gs belonged to lineage B.1.177. In addition, the viral sequences from patients Cm and Ba were classified into lineage B.1.1.

Furthermore, a clade classification by Nextstrain was also obtained. The results showed that the viral sequences from patients As, Da, Em, Fs and Gs were all associated to clade 20A, while Ba and Cm belonged to clade 20B. The mutation shared between patients Fs and Gs was observed in both a branch of clade 20B as well as in clade 20E.EU1. However, it must be noted that the latter clade is defined by the presence of A222V amino acidic variation in the spike protein, that, as already mentioned, was absent in these patients. In conclusion, there was no association between a specific viral lineage/clade and a specific patient group.

**Fig 2:**
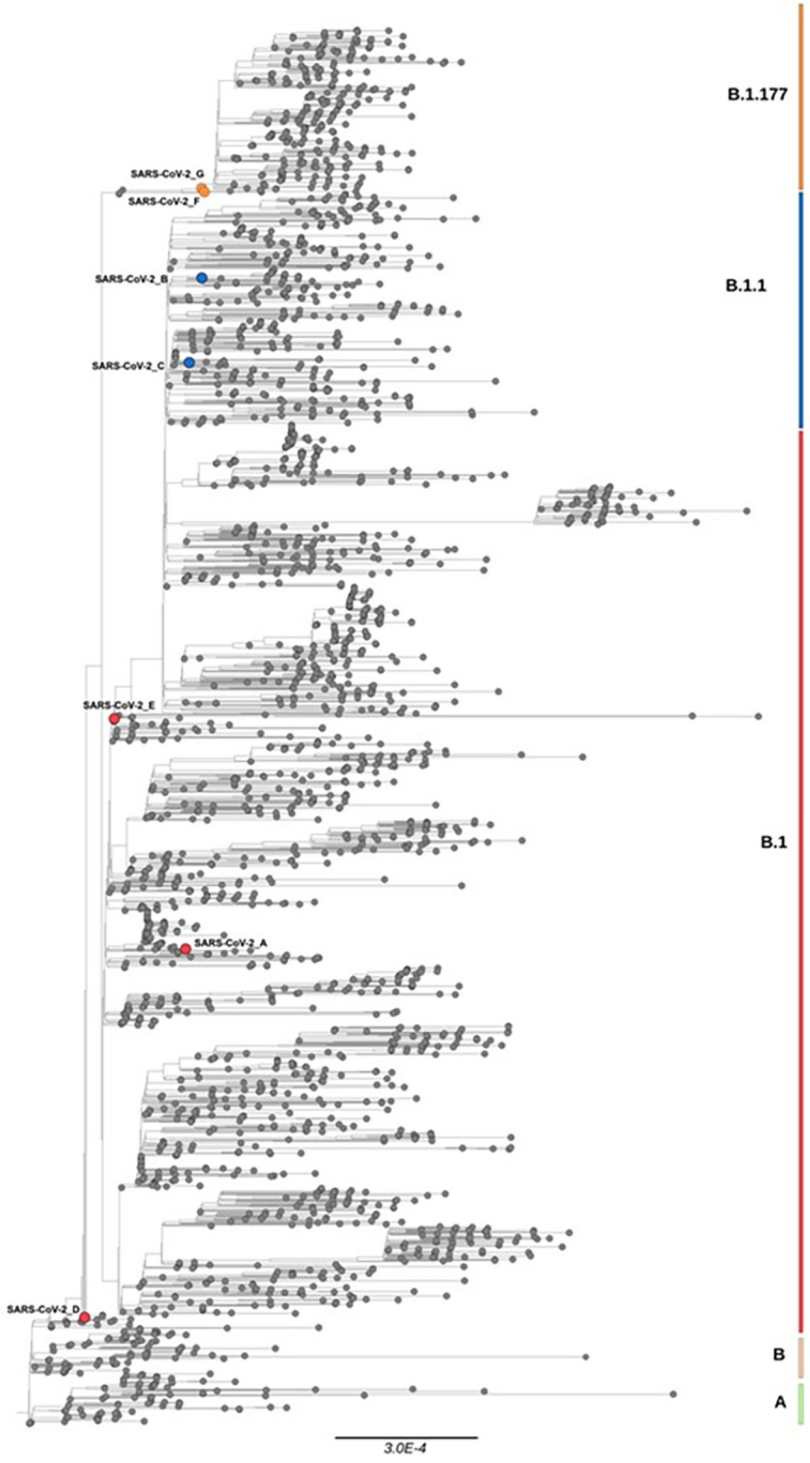
Maximum-likelihood (ML) tree of SARS-CoV-2 sequences. The ML tree includes 2463 SARS-CoV-2 sequences retrieved globally from GISAID database until December 2021 and 7 SARS-CoV-2 sequences evaluated in this study represented as dots. SARS-CoV-2 sequences of this study are colored by lineages: B.1 in red; B.1.1 in blue; B.1.177 in orange; all the other world sequences in black.

Finally, in order to accurately assess possible evolutionary relationships on a global scale among these seven Italian SARS-CoV-2 sequences, a maximum likelihood (ML) tree was implemented. The dataset utilized in this analysis consists of 2463 global sequences collected from the beginning of pandemic until December 2021 and belonging to different lineages: A, B, B.1, B.1.1, B.1.177. As shown in Fig 2, the two sequences found in patients Fs and Gs, two of the severely symptomatic ones, gave rise to the B.1.177 cluster, while the remaining sequences from the other patients were located in accordance with their lineage but are scattered across the phylogenetic tree. Interestingly, phylogenetic analysis revealed that the GISAID sequences EPI_ISL_539548 and EPI_ISL_3716577, located at the beginning of B.1.177 branch before patients Fs and Gs, derived from symptomatic and hospitalized patients.

## Discussion

COVID-19 is mainly a respiratory syndrome. However, since the beginning of the disease in a subset of individuals extra-respiratory symptoms/complications could arise. Among those, neurological manifestations, ranging from olfactory dysfunction to any neurologic manifestation, with the most common being encephalopathy, have been of great concern since the beginning of the pandemic [11, 38]. The mechanisms accounting for the onset of neurological symptoms/complications in certain COVID-19 patients is still matter of study, especially in terms of the role directly played by the virus [11, 28, 38, 39].

The aim of this study was to analyze whether SARS-CoV-2 genomes directly retrieved from the nasopharyngeal swabs of patients characterized by severe CNS manifestations displayed specific and common signatures not shared with the ones obtained from asymptomatic/mild symptomatic subjects. In particular, seven SARS-CoV-2 positive individuals were enrolled between March and November 2020. These patients were either fully asymptomatic or experienced variable neurological manifestations, including three subjects admitted to the intensive care unit of the Padua Hospital due to severe encephalopathy. In this cohort of patients, four were between 25 and 35 years old, one around 55 and two above 70 years old. Neurological symptoms and complications displayed by the selected subjects are reported in the literature among the most frequent COVID19-related neurological manifestations [13, 39]. All patients in the most severe neurological conditions presented important respiratory symptoms, such as interstitial pneumonia, and were intubated. This finding supports the notion that patients with severe disease are more likely to develop neurological disorders [40, 41]. Finally, and not surprisingly [42, 43], the three hospitalized patients were also the oldest of this cohort and two out of three were males.

Data demonstrate that SARS-CoV-2 can access the CNS and can potentially damage the brain tissues by directly lysing infected cells and by triggering a localized inflammatory response [11, 28]. On the other hand, SARS-CoV-2 replication in the lungs stimulates a cytokine storm that could also affect the CNS, even in the absence of a direct brain invasion by the virus [28, 29]. The two SARS-CoV-2 envelope proteins S and E could play a role in both these mechanisms. Indeed, while the S protein is the main viral tropism determinant [10, 11], the E protein is involved in different steps of viral replication and in regulating viral/cell host interactions [44]. Studies focused on SARS-CoV-1, in particular, have shown that E protein is a virulence factor involved in the activation of various inflammatory pathways [45]. Of note, the E genes of SARS-CoV-1 and SARS-CoV-2 are highly identical. Based on these evidence, we started by Sanger sequencing the S and E genes of SARS-CoV-2 genomes directly extracted from the nasopharyngeal swabs of the enrolled cohort. However, with the exception of the well-known and expected D614G mutation [36], present in the S gene and common to all the analyzed sequences, no other mutations were detected in either the S or E genes.

Next, the same viral RNA extracts underwent whole genome analysis, obtaining a classification of the SARS-CoV-2s under investigation into three different lineages: B.1, B.1.177 and B1.1. More specifically, viral sequences obtained from patients As, Da and Em were linked to the B.1 lineage, which has been circulating since mid-January 2020, and is characterized by 4 predominant mutations. These mutations include D614G and P323L that have been observed in all the analyzed patients. The B.1 lineage was the most prevalent lineage in Europe during 2020 (Pangolin Cov-lineages: https://cov-lineages.org/resources/pangolin.html accessed on the 15^th^ of March 2021). The viral sequences from patients Fs and Gs were linked to the B.1.177 lineage, which is a sub-lineage of the B.1 lineage. These sequences shared the same 4 mutations as before, but with the addition of A220V in the nucleocapsid N protein. The B.1.177 lineage had spread mainly in Europe during summer 2020 and is slowly disappearing (Pangolin Cov-lineages: https://cov-lineages.org/resources/pangolin.html).

The viral genomes sequenced from patients Cm and Ba were associated to the B.1.1 lineage, which derives from lineage B.1. These viral sequences were characterized by the additional presence of 3 consecutive SNVs at position 28881-2-3. This lineage was also prevalent in Europe, specifically in England, circulating since mid-February (Pangolin Cov-lineages: https://cov-lineages.org/resources/pangolin.html). The Nextstrain classification into clades showed that the viral sequences from patients As and Da, together with patients Em, Fs and Gs were all classified into clade 20A, while those from patients Ba and Cm were classified into clade 20B. The 4 most common mutations shared by the B.1, B.1.177, B.1.1 lineages were 4 SNVs: C241T, C3037T, C14408T, A23403G. These 4 SNVs that have been widely described in literature [46, 47], appeared when the virus started circulating outside of China, and are widely distributed in Europe and in the USA. Indeed, since the beginning of March 2020, when, according to the databases, these mutations were present in 10% of the analyzed sequences, their frequency increased exponentially, reaching 78% by the end of May 2020 [48] and overall constituted the dominant haplotype in Europe during year 2020. Indeed, these mutations, which are located far apart from each other in the genome, have a strong allelic association, likely due to different factors, as a founder effect or a gain in viral fitness [49].

C241T is a non-coding mutation located in the 5’ UTR, and even if it does not cause changes in the amino acid sequence, it could still lead to a relevant change in the secondary structure of the RNA or/and modify the repertoire of interactions with viral and cellular proteins, thus affecting the RNA replication or the speed of the infection cycle [28]. C3037T is a single nucleotide substitution in position 3037 of the RNA genome, located in the nsp3 gene. It is a silent, synonymous, mutation named F924; it is located in the nsp3 protein, which is a phosphodiesterase. Although silent, this mutation may affect the RNA secondary structure, and therefore the interaction with other proteins [36].

C14408T is a non-synonymous mutation in the nsp12, which is the RNA-dependent RNA-polymerase [50]. This single nucleotide variation leads to an amino acid substitution from a proline (P) to a leucine (L), in position 323 of the nsp12, which in turn is a P4715L substitution in the ORF1ab. This mutation is located outside the catalytic site, specifically at the interface domain of the protein, that is responsible for the interaction with other proteins [51]. According to literature, this mutation could have multiple effects. Not only it could cause an increased interaction with the SARS-CoV-2 non-structural protein nsp8, which is a processivity factor; but it could also alter the interaction with the viral nsp14, an exonuclease responsible for the proof-reading activity of the polymerase. This way, it could affect the fidelity of replication and be responsible for a higher mutational rate [50]. Furthermore, given its hydrophobicity, leucine tends to remain inside the secondary structure, rather than being exposed to the outside, as proline does; thus, the substitution could cause the loss of one of the nsp12 epitopes leading to antibodies escape [52].

A23403G single nucleotide variation is in the spike-encoding gene. This SNV causes an amino acid change from a hydrophilic and negatively charged aspartic acid (D) to hydrophobic and uncharged glycine (G), therefore it is called D614G. In literature, this mutation is reported to be associated with increased virus infectivity and transmissibility [48, 53]. However, the reason for this increase remains to be answered. On the one hand, it is thought to be related to an increased affinity for the angiotensin converting enzyme 2 (ACE2) receptor which, in turns, increases the entry efficiency of the virus [54]. On the other hand, it seems to be related to a more efficient incorporation of the spike in the newly formed virions, due to its greater stability and reduced shedding of the S1 subunit upon cleavage [55]. It must be noted that, overall, this set of four mutations was not linked to either a greater disease severity, in terms of hospitalization rate, nor to a reduced antibody neutralization [48].

Viral sequences from the asymptomatic patient Ba and the mild symptomatic patient Cm were characterized not only by the four mutations described above, but also by a variation of three nucleotides at position 28881-2-3 [56]. This three-nucleotide variation from GGG to AAC is located within the gene encoding for the viral nucleocapsid N protein. These three SNVs have a strong allelic association and lead to an amino acid change at position 50–51 from arginine-glycine to lysin-arginine, thus bringing together two basic and polar amino acids, which could create a stronger binding between the N protein and the viral RNA. These mutations are expected to affect viral infectivity, due to the replacement of an arginine-glycine by a lysine-arginine in a serine-rich motif of the nucleoprotein [57]. This triplet characterizes most of the sequences deposited in GISAID belonging to the B.1.1 lineage first reported in late February 2020. This lineage was predominant until June 2020, when it started rapidly decreasing in its frequency [37]. Indeed, there was a reversion of this mutated triplet into the original one, and a parallel spread of a new variant characterized by 2 spike mutations associated with an A220V mutation of the nucleocapsid N protein. However, the mutated triplet reappeared in the most recent variants, and it can be found also in sequences belonging to the Omicron variant (https://www.gisaid.org/ accessed on May 17^th^ 2022).

Of those patients with the most severe symptoms, the viral RNA obtained from patients Fs and Gs showed the A220V amino acid change of the N protein. This mutation has a similar distribution pattern to the A222V spike mutation; these two mutations are generally associated and define the 20A.EU1 clade. This variant dated back to summer 2020 when it appeared in the Netherlands and then in Spain and spread throughout Europe, and with a lower frequency in the rest of the world [37, 56]. The A220V in N protein propagated rapidly, associated with two spike mutations (L18F and A222V), in the second wave of the pandemic. However, in our cohort, these two spike mutations were absent in all analyzed patients. On the one hand, the A220V variation was only present in two severely symptomatic patients and neither in the controls, nor in the mild symptomatic patients. On the other hand, the A220V mutation was not shared by the third severely symptomatic patient As. Of note, the same mutation was found in two GISAID sequences (EPI_ISL_539548 and EPI_ISL_3716577) obtained from symptomatic patients.

The symptomatic patient As, asymptomatic Ba and mild-symptomatic Cm had unique mutations in their viral sequence. The alignment with sequences in the database showed that all these mutations were rare, with a frequency below 1% and none of them was phenotypically characterized. Therefore, if the frequency of these non-synonymous mutations will increase, it could be important to evaluate whether they may affect the corresponding protein function. On the other side, none of these “novel” mutations was shared between the three patients.

In conclusion, while interesting mutations were found in the viral genome under study, from the overall analysis no correlation is evident between symptoms and viral sequence features. Furthermore, the MRI performed on the patients excludes the possibility of a brain localized inflammatory response. In fact, the MRI of each patient was always negative, with exception of one patient (Fs), where there was a concomitant ischemic episode. Hence, overall, our data support the notion that COVID-19 neurological manifestations are linked to the individual inflammatory response, rather than to specific signature of the viral genome [58]. The main limit of this pilot study is surely the small number of enrolled patients. However, on one hand, and as mentioned before, these data are worth of attention, as they have been obtained by whole genome sequencing performed on viral RNA directly extracted from the nasopharyngeal swabs of the enrolled patients, without viral amplification in cell culture. This procedure is recommended by the WHO to avoid accumulation of extra mutations not reflecting the *in vivo* situation [59]. On the other hand, the sampled population is heterogeneous in terms, for instance, of age and sex. Indeed, despite the limited number of patients, we found an association between neurological and severe respiratory symptoms and between symptom severity in general, and age and sex, as reported in the literature [42, 60]. Thus, the reported results are worth of attention and deserve further validation in larger cohorts of patients.

## References

[1] C S G of the International. The species Severe acute respiratory syndrome-related coronavirus: classifying 2019-nCoV and naming it SARS-CoV-2. Nat Microbiol. 2020;5(4):536. doi: 10.1038/s41564-020-0695-z 32123347 PMC7095448

[2] Zhou P, Yang XL, Wang XG, Hu B, Zhang L, Zhang W, et al. A pneumonia outbreak associated with a new coronavirus of probable bat origin. Nature. 2020;579(7798):270–273. doi: 10.1038/s41586-020-2012-7 32015507 PMC7095418

[3] Ashour HM, Elkhatib WF, Rahman M, Elshabrawy HA. Insights into the recent 2019 novel coronavirus (SARS-CoV-2) in light of past human coronavirus outbreaks. Pathogens. 2020;9(3):186. doi: 10.3390/pathogens9030186 32143502 PMC7157630

[4] Ziebuhr J. The coronavirus replicase. vol. 287. Springer; 2005. 10.1007/3-540-26765-4_3 PMC7121973 15609509

[5] Alanagreh L, Alzoughool F, Atoum M. The human coronavirus disease COVID-19: its origin, characteristics, and insights into potential drugs and its mechanisms. Pathogens. 2020;9(5):331. 10.3390/pathogens9050331 PMC7280997 32365466

[6] Romano M, Ruggiero A, Squeglia F, Maga G, Berisio R. A structural view of SARS-CoV-2 RNA replication machinery: RNA synthesis, proofreading and final capping. Cells. 2020;9(5):1267. doi: 10.3390/cells9051267 32443810 PMC7291026

[7] Alteri C, et al. Genomic epidemiology of SARS-CoV-2 reveals multiple lineages and early spread of SARS-CoV-2 infections in Lombardy, Italy. Nat Commun. 2021;12(1):43. 33469026 10.1038/s41467-020-20688-x PMC7815831

[8] Tao K, Tzou PL, Nouhin J, Gupta RK, de Oliveira T, Kosakovsky Pond SL, et al. The biological and clinical significance of emerging SARS-CoV-2 variants. Nat Rev Genet. 2021;22(12):757–773. doi: 10.1038/s41576-021-00408-x 34535792 PMC8447121

[9] Ghosh N, Nandi S, Saha I. A Review on evolution of emerging SARS-CoV-2 variants based on spike glycoprotein. Int Immunopharmacol. 2022;105:108565. doi: 10.1016/j.intimp.2022.108565 35123183 PMC8799522

[10] Harrison AG, Lin T, Wang P. Mechanisms of SARS-CoV-2 transmission and pathogenesis. Trends Immunol. 2020;41(12):1100–1115. doi: 10.1016/j.it.2020.10.004 33132005 PMC7556779

[11] Haidar MA, Shakkour Z, Reslan MA, Al-Haj N, Chamoun P, Habashy K, et al. SARS-CoV-2 involvement in central nervous system tissue damage. Neural Regen Res. 2022;17(6):1228. doi: 10.4103/1673-5374.327323 34782556 PMC8643043

[12] Baig AM. Neurological manifestations in COVID-19 caused by SARS-CoV-2. CNS Neurosci Ther. 2020;26(5):499. doi: 10.1111/cns.13372 32266761 PMC7163592

[13] Collantes M E V, Espiritu A I, Sy M C C, Anlacan V M M, Jamora R D G. Neurological manifestations in COVID-19 infection: a systematic review and meta-analysis. Can J Neurol Sci. 2021; 48(1), 66–76. doi: 10.1017/cjn.2020.146 32665054 PMC7492583

[14] Lechien JR, Chiesa-Estomba CM, De Siati DR, Horoi M, Le Bon SD, Rodriguez A, et al. Olfactory and gustatory dysfunctions as a clinical presentation of mild-to-moderate forms of the coronavirus disease (COVID-19): a multicenter European study. Eur Arch Oto-Rhino-L. 2020;277(8):2251–2261. doi: 10.1007/s00405-020-05965-1 32253535 PMC7134551

[15] Brann DH, Tsukahara T, Weinreb C, Lipovsek M, Van den Berge K, Gong B, et al. Non-neuronal expression of SARS-CoV-2 entry genes in the olfactory system suggests mechanisms underlying COVID-19-associated anosmia. Sci Adv. 2020;6(31):eabc5801. doi: 10.1126/sciadv.abc5801 32937591 PMC10715684

[16] Pilotto A, Odolini S, Masciocchi S, Comelli A, Volonghi I, Gazzina S, et al. Steroid-responsive encephalitis in coronavirus disease 2019. Ann Neurol. 2020;88(2):423–427. doi: 10.1002/ana.25783 32418288 PMC7276848

[17] Muccioli L, Pensato U, Cani I, Guarino M, Cortelli P, Bisulli F. COVID-19-associated encephalopathy and cytokine-mediated neuroinflammation. Ann Neurol. 2020;88(4):860–861. doi: 10.1002/ana.25855 32715524

[18] Paterson RW, Brown RL, Benjamin L, Nortley R, Wiethoff S, Bharucha T, et al. The emerging spectrum of COVID-19 neurology: clinical, radiological and laboratory findings. Brain. 2020;143(10):3104–3120. doi: 10.1093/brain/awaa240 32637987 PMC7454352

[19] Bergmann CC, Lane TE, Stohlman SA. Coronavirus infection of the central nervous system: host–virus stand-off. Nat Rev Microbiol. 2006;4(2):121–132. doi: 10.1038/nrmicro1343 16415928 PMC7096820

[20] Desforges M, Le Coupanec A, Dubeau P, Bourgouin A, Lajoie L, Dubé M, et al. Human coronaviruses and other respiratory viruses: underestimated opportunistic pathogens of the central nervous system? Viruses. 2020;12(1):14. 10.3390/v12010014 PMC7020001 31861926

[21] Wu Y, Xu X, Chen Z, Duan J, Hashimoto K, Yang L, et al. Nervous system involvement after infection with COVID-19 and other coronaviruses. Brain Behav Immun. 2020;87:18–22. doi: 10.1016/j.bbi.2020.03.031 32240762 PMC7146689

[22] Pujadas E, Beaumont M, Shah H, Schrode N, Francoeur N, Shroff S, et al. Molecular Profiling of Coronavirus Disease 2019 (COVID-19) Autopsies Uncovers Novel Disease Mechanisms. Am J Pathol. 2021;191(12):2064–2071. doi: 10.1016/j.ajpath.2021.08.009 34506752 PMC8423774

[23] Chen R, Wang K, Yu J, Howard D, French L, Chen Z, et al. The spatial and cell-type distribution of SARS-CoV-2 receptor ACE2 in the human and mouse brains. Front Neurol. 2021;11:1860. doi: 10.3389/fneur.2020.573095 33551947 PMC7855591

[24] Deguchi S, Serrano-Aroca Á, Tambuwala MM, Uhal BD, Brufsky AM, Takayama K. SARS-CoV-2 research using human pluripotent stem cells and organoids. Stem Cells Transl Med. 2021;10(11):1491–1499. doi: 10.1002/sctm.21-0183 34302450 PMC8550698

[25] Zhang BZ, Chu H, Han S, Shuai H, Deng J, Hu Yf, et al. SARS-CoV-2 infects human neural progenitor cells and brain organoids. Cell Res. 2020;30(11):60131. doi: 10.1038/s41422-020-0390-x 32753756 PMC7399356

[26] Matschke J, Lütgehetmann M, Hagel C, Sperhake JP, Schröder AS, Edler C, et al. Neuropathology of patients with COVID-19 in Germany: a post-mortem case series. Lancet Neurol. 2020;19(11):919–929. doi: 10.1016/S1474-4422(20)30308-2 33031735 PMC7535629

[27] Virhammar J, Kumlien E, Fällmar D, Frithiof R, Jackmann S, Sköld MK, et al. Acute necrotizing encephalopathy with SARS-CoV-2 RNA confirmed in cerebrospinal fluid. Neurology. 2020;95(10):445–449. doi: 10.1212/WNL.0000000000010250 32586897 PMC7538220

[28] Kumar A. COVID-19 Current challenges and future perspectives. Betham Books 2021. 8: 76–100.

[29] Tay MZ, Poh CM, Rénia L, MacAry PA, Ng LF. The trinity of COVID-19: immunity, inflammation and intervention. Nat Rev Immunol. 2020;20(6):363–374. doi: 10.1038/s41577-020-0311-8 32346093 PMC7187672

[30] Li C, et al. Highly sensitive and full-genome interrogation of SARS-CoV-2 using multiplexed PCR enrichment followed by next-generation sequencing. BioRxiv. 2020; Forthcoming.

[31] Al Khatib HA, Benslimane FM, Elbashir IE, Coyle PV, Al Maslamani MA, Al-Khal A, et al. Within-host diversity of SARS-CoV-2 in COVID-19 patients with variable disease severities. Front Cell Infect Microbiol. 2020; 534. doi: 10.3389/fcimb.2020.575613 33123498 PMC7572854

[32] Rambaut A, Holmes EC, O’Toole Á, Hill V, McCrone JT, Ruis C, et al. A dynamic nomenclature proposal for SARS-CoV-2 lineages to assist genomic epidemiology. Nat Microbiol. 2020;5(11):1403–1407. doi: 10.1038/s41564-020-0770-5 32669681 PMC7610519

[33] Katoh K, Rozewicki J, Yamada KD. MAFFT online service: multiple sequence alignment, interactive sequence choice and visualization. Brief Bioinformatics. 2019;20(4):1160–1166. doi: 10.1093/bib/bbx108 28968734 PMC6781576

[34] Larsson A. AliView: a fast and lightweight alignment viewer and editor for large datasets. Bioinformatics. 2014;30(22):3276–3278. doi: 10.1093/bioinformatics/btu531 25095880 PMC4221126

[35] Nguyen LT, Schmidt HA, Von Haeseler A, Minh BQ. IQ-TREE: a fast and effective stochastic algorithm for estimating maximum-likelihood phylogenies. Mol Biol Evol. 2015;32(1):268–274. doi: 10.1093/molbev/msu300 25371430 PMC4271533

[36] Mercatelli D, Giorgi FM. Geographic and genomic distribution of SARS-CoV-2 mutations. Front Microbiol. 2020; 1800. doi: 10.3389/fmicb.2020.01800 32793182 PMC7387429

[37] Vilar S, Isom DG. One year of SARS-CoV-2: how much has the virus changed? Biology. 2021;10(2):91. doi: 10.3390/biology10020091 33530355 PMC7911924

[38] Hingorani K S, Bhadola S, Cervantes-Arslanian A M. COVID-19 and the Brain. Trends cardiovasc. Med. 2022; doi: 10.1016/j.tcm.2022.04.004 35461991 PMC9022395

[39] Xu Y, Zhuang Y, Kang L. A review of neurological involvement in patients with SARS-CoV-2 infection. Med. Sci. Monit. 2021; 27: e932962–1. doi: 10.12659/MSM.932962 34145211 PMC8221270

[40] Mao L et al. Neurological manifestations of hospitalized patients with COVID-19 in Wuhan, China: A retrospective case series study (February 24, 2020). JAMA Neurol. 2020; 77(6): 1–9. 10.1001/jamaneurol.2020.1127 PMC7149362 32275288

[41] Aghagoli G, Gallo Marin B, Katchur N J, Chaves-Sell F, Asaad W F, Murphy S A. Neurological involvement in COVID-19 and potential mechanisms: a review. Neurocritical care. 2021; 34(3): 1062–1071. doi: 10.1007/s12028-020-01049-4 32661794 PMC7358290

[42] Tazerji SS et al. Global data analysis and risk factors associated with morbidity and mortality of COVID-19. Gene Rep. 2022; 26: 101505. doi: 10.1016/j.genrep.2022.101505 35071820 PMC8761036

[43] Ellul M A et al. Neurological associations of COVID-19. The Lancet Neurology, 2020; 19(9): 767–783. doi: 10.1016/S1474-4422(20)30221-0 32622375 PMC7332267

[44] Zhu Y et al. Interactions of Severe Acute Respiratory Syndrome Coronavirus 2 Protein E With Cell Junctions and Polarity PSD-95/Dlg/ZO-1-Containing Proteins. Front Microbiol. 2022; 13: 829094. doi: 10.3389/fmicb.2022.829094 35283834 PMC8909127

[45] Morales L, Oliveros JC, Enjuanes L, Sola I. Contribution of Host miRNA-223-3p to SARS-CoV-Induced Lung Inflammatory Pathology. mBio. 2022; 13(2): e0313521. doi: 10.1128/mbio.03135-21 35229638 PMC8941895

[46] Weber S, Ramirez C, Doerfler W. Signal hotspot mutations in SARS-CoV-2 genomes evolve as the virus spreads and actively replicates in different parts of the world. Virus Res. 2020;289:198170. doi: 10.1016/j.virusres.2020.198170 32979477 PMC7513834

[47] Yang HC, Chen Ch, Wang JH, Liao HC, Yang CT, Chen CW, et al. Analysis of genomic distributions of SARS-CoV-2 reveals a dominant strain type with strong allelic associations. Proc Natl Acad Sci USA. 2020;117(48):30679–30686. doi: 10.1073/pnas.2007840117 33184173 PMC7720151

[48] Korber B, Fischer WM, Gnanakaran S, Yoon H, Theiler J, Abfalterer W, et al. Tracking changes in SARS-CoV-2 spike: evidence that D614G increases infectivity of the COVID-19 virus. Cell. 2020;182(4):812–827. doi: 10.1016/j.cell.2020.06.043 32697968 PMC7332439

[49] van Dorp L, Richard D, Tan C, Shaw LP, Acman M, Balloux F. No evidence for increased transmissibility from recurrent mutations in SARS-CoV-2. Nat Commun. 2020;11(1):5986. doi: 10.1038/s41467-020-19818-2 33239633 PMC7688939

[50] Pachetti M, Marini B, Benedetti F, Giudici F, Mauro E, Storici P, et al. Emerging SARS-CoV-2 mutation hot spots include a novel RNA-dependent-RNA polymerase variant. J Transl Med. 2020;18(1):179. doi: 10.1186/s12967-020-02344-6 32321524 PMC7174922

[51] Kannan SR, Spratt AN, Quinn TP, Heng X, Lorson CL, onnerborg A, et al. Infectivity of SARS-CoV-2: there is something more than D614G? J Neuroimmune Pharmacol. 2020;15(4):574–577. doi: 10.1007/s11481-020-09954-3 32930936 PMC7490321

[52] Gupta AM, Chakrabarti J, Mandal S. Non-synonymous mutations of SARS-CoV-2 leads epitope loss and segregates its variants. Microbes Infect. 2020;22(10):598–607. doi: 10.1016/j.micinf.2020.10.004 33049387 PMC7547839

[53] Plante JA, Liu Y, Liu J, Xia H, Johnson BA, Lokugamage KG, et al. Spike mutation D614G alters SARS-CoV-2 fitness. Nature. 2021;592(7852):116–121. doi: 10.1038/s41586-020-2895-3 33106671 PMC8158177

[54] Ozono S, Zhang Y, Ode H, Sano K, Tan TS, Imai K, et al. SARS-CoV-2 D614G spike mutation increases entry efficiency with enhanced ACE2-binding affinity. Nat Commun. 2021;12(1):848. doi: 10.1038/s41467-021-21118-2 33558493 PMC7870668

[55] Zhang L, Jackson CB, Mou H, Ojha A, Peng H, Quinlan BD, et al. SARS-CoV-2 spike-protein D614G mutation increases virion spike density and infectivity. Nat Commun. 2020;11(1):1–9. 33243994 10.1038/s41467-020-19808-4 PMC7693302

[56] Hodcroft EB, Zuber M, Nadeau S, Vaughan TG, Crawford KH, Althaus CL, et al. Spread of a SARS-CoV-2 variant through Europe in the summer of 2020. Nature. 2021;595(7869):707–712. doi: 10.1038/s41586-021-03677-y 34098568

[57] Ayub M, et al. Reporting two SARS-CoV-2 strains based on a unique trinucleotide-bloc mutation and their potential pathogenic difference. Preprintsorg. 2020; Forthcoming.

[58] Sulzer D, Antonini A, Leta V, Nordvig A, Smeyne RJ, Goldman JE, et al. COVID-19 and possible links with Parkinson’s disease and parkinsonism: from bench to bedside. NPJ Parkinson’s Dis. 2020;6(1):1–10. doi: 10.1038/s41531-020-00123-0 32885037 PMC7441399

[59] World Health Organization, Genomic Sequencing of SARS-CoV-2: A Guide to Implementation for Maximum Impact on Public Health. Geneva 2021.

[60] Pijls BG et al. Temporal trends of sex differences for COVID-19 infection, hospitalisation, severe disease, intensive care unit (ICU) admission and death: a meta-analysis of 229 studies covering over 10M patients. F1000Res. 2022; 11(5): 5. doi: 10.12688/f1000research.74645.1 35514606 PMC9034173

